# Indications and Outcomes of Parotidectomy in Saudi Children: Experience From Two Tertiary Centers

**DOI:** 10.7759/cureus.19587

**Published:** 2021-11-15

**Authors:** Ahmad Albosaily, Turki Aldrees, Saleh Aldhahri, Khalid Al-Qahtani

**Affiliations:** 1 Department of Otolaryngology, King Saud University, Riyadh, SAU; 2 Department of Otolaryngology, Prince Sattam Bin Abdulaziz University, Alkharj, SAU

**Keywords:** mucoepidermoid carcinoma, salivary gland, pleomorphic adenoma, children, parotid surgery, parotidectomy, pediatric

## Abstract

Introduction

Children can present with a wide variety of parotid diseases. However, most of them do not require surgical treatment. The indications of parotid surgery in children may differ from those in adults. In this study, we aim to review the indications and outcomes of parotidectomy in our pediatric population.

Methods

Retrospective review of the medical records of patients who underwent parotid surgery at age <18 years in two tertiary centers over a 14-year period.

Results

A total of 18 parotidectomies were performed on 18 patients with a mean age of 13.5 years. All patients presented with a parotid mass. The most common procedure was superficial parotidectomy followed by total parotidectomy. Ten patients were diagnosed with a benign parotid disease (55.6%). The most common benign disease was pleomorphic adenoma. There were eight cases of parotid malignancy constituting 44.4% of all patients and 57% of patients presenting in the age range of 12-17 years. Mucoepidermoid carcinoma was the most common malignancy (six patients). Fine needle aspiration biopsy was performed in 12 children with a sensitivity, specificity, and overall accuracy of 62.5%, 50%, and 58.6%, respectively. The most common complication was temporary facial weakness (33.3%) which resolved in a mean time of 2.5 months. No disease recurrences were identified.

Conclusions

Parotidectomy is uncommon in the pediatric age group. Children with a solid parotid tumor have a higher risk of parotid malignancy when compared to adults. A parotid mass presenting in the second decade of life is more likely to be malignant than benign.

## Introduction

Parotid lesions in children are uncommon and can represent a wide spectrum of congenital, inflammatory, and neoplastic conditions, making diagnosis and treatment of these lesions challenging [1,2]. Furthermore, lesions requiring parotidectomy in children are even rarer. Only 5% of all salivary gland neoplasms, with the majority involving the parotid gland, occur in patients under 18 years of age [3].

In this study, we aim to present our 14-year experience from two tertiary centers to identify the indications and complications of parotidectomy in patients under the age of 18 years in the Saudi population.

## Materials and methods

The study was approved by the institutional review board (IRB) of the college of medicine, King Saud University (approval No. E-19-3901). We con­ducted a retrospective review of the medical records of patients who underwent parotid surgery at age <18 years in two tertiary centers (King Abdulaziz University Hospital and King Fahad Medical City) in Riyadh, Saudi Arabia, from January 2007 to December 2020. Data on gender, age, presentation, investigations, surgery and its complications, pathological diagnosis, adjuvant treatments, clinical follow-up, and disease recurrence were collected. The development of facial weakness after surgery was recorded as temporary when weakness resolved completely upon follow-up, while permanent weakness was defined as the failure to regain normal movement with­in 12 months. Patients who underwent parotid duct ligation, incision, and drainage of parotid abscess or biopsy of parotid lesions were excluded. IBM statistics program for social science (SPSS) software version 22.0 (IBM Inc., Armonk, New York) was used to conduct the statistical analysis. Descriptive statistical data are presented as means and standard deviations (SD), ranges and frequencies, and percentages. Crosstabs were utilized to present the relationship between variables.

## Results

Out of 289 parotidectomies performed during the study period, 18 parotidectomies (6.23%) were performed on 18 pediatric patients. There were five males and 13 females with a mean age of 13.5±4.018 years and a mean follow-up period of 36.83±34.73 months.

All patients presented with a parotid mass, with a mean duration of 16.29±16.84 months. Twelve patients (66.7%) had fine-needle aspiration (FNA), five patients were referred with histological diagnosis after incisional biopsy or incomplete surgery, and one patient had no preoperative biopsy. There were 11 superficial parotidectomies, six total parotidectomies, and one radical parotidectomy.

Ten patients (55.6%) were diagnosed with benign lesions with a mean age of 11.9±4.6 years. Pleomorphic adenoma was the most common benign lesion (40%). Other benign lesions were hemangioma, aggressive fibromatosis, intranodal palisaded myofibroblastoma (IPM), sialadenitis, branchial cyst, and benign lymph node hyperplasia (Table 1). The male to female ratio among patients with benign lesions was 1:4.

**Table 1:** Final pathological diagnosis

Diagnosis	Frequency	Percentage
Inflammatory/congenital		
Sialadenitis	1	5.6%
Branchial cyst	1	5.6%
Solid tumors		
Benign		
Pleomorphic adenoma	4	22.2%
Benign lymph node hyperplasia	1	5.6%
Hemangioma	1	5.6%
Aggressive Fibromatosis	1	5.6%
Intranodal palisaded myofibroblastoma	1	5.6%
Malignant		
High-grade mucoepidermoid carcinoma	5	27.8%
Low-grade mucoepidermoid carcinoma	1	5.6%
Lymphoma	2	11.1%

Eight patients (44.4%) were diagnosed with malignant lesions with a mean age of 15.5±1.93 years (Range 12-17 years). Mucoepidermoid carcinoma (MEC) was the most common malignancy (six cases), followed by lymphoma (two cases) (Table 1). The male to female ratio among patients with malignant lesions was 1:1.67. Four patients with mucoepidermoid carcinoma received adjuvant intensity-modulated radiotherapy (IMRT). No radiotherapy-related long-term side effects were recorded during follow-up. All patients with epithelial malignancies were followed up radiologically using computed tomography (CT) or magnetic resonance imaging (MRI) at different intervals, except one patient who lost follow-up nine months after surgery. No local disease recurrences were recorded. The details of epithelial malignancies are summarized in Table 2.

**Table 2:** Summary of epithelial parotid malignancies F: female, M: male, L: left, R: right, SP: superficial parotidectomy, TP: total parotidectomy, HG MEC: high-grade mucoepidermoid carcinoma, LG MEC: low-grade mucoepidermoid carcinoma, IMRT: intensity-modulated radiotherapy

Age (years)	Gender	Side	Surgery type	Diagnosis	Adjuvant treatment	Follow-up (months)	Status at last follow-up
14	F	L	SP	HG MEC	None	92	Alive, no recurrence
14	F	R	TP	HG MEC	IMRT	88	Alive, no recurrence
16	M	R	TP	HG MEC	IMRT	21	Alive, no recurrence
17	F	R	TP	LG MEC	IMRT	67	Alive, no recurrence
17	M	L	TP	HG MEC	IMRT	9	Lost follow up
17	F	R	TP	HG MEC	None	76	Alive, no recurrence

The fine-needle aspiration cytology (FNAC) showed a sensitivity of 62.5%, specificity of 50%, and an overall accuracy of 58.4% for the diagnosis of benign and malignant tumors (Table 3).

**Table 3:** Results of fine-needle aspiration cytology and histopathologic examination FNAC was performed in 12 patients. FNAC: fine-needle aspiration cytology

		Histopathological results	
		Benign (n=8)	Malignant (n=4)
FNAC results	Benign (n=6)	5	1
	Malignant (n= 2)	0	2
	Inconclusive (n=4)	3	1

The most common postoperative complication was facial weakness. Six patients (33.3%) had temporary facial weakness, with a mean resolution time of 2.50±1.22 months. One patient (5.5%) had inadvertent permanent weakness of the marginal branch due to tumor adherence and difficult dissection while one patient with aggressive fibromatosis (5.5%) had expected complete permanent facial paralysis as a result of facial nerve sacrifice (radical parotidectomy). Other complications were: periauricular numbness (22.2%), Frey's syndrome (12.5%), hematoma (5.6%), and hypertrophic scarring (5.6%).

## Discussion

Parotidectomy is an uncommon procedure. It is even less commonly indicated in the pediatric age group [1,3]. In our study, it only constituted around 6% of all parotidectomies performed. Nevertheless, this is in no way reflective of the prevalence of parotid lesions in this age group. Some inflammatory and Congenital lesions such as hemangiomas and vascular malformations are relatively prevalent; however, surgery is not their primary modality of treatment [2].

The differential diagnoses of pediatric parotid masses are more variable than those in adults [2]. This makes the diagnosis more challenging and calls for a comprehensive workup before proceeding to surgery. Ultrasonography is radiation-free and doesn’t require sedation, and can differentiate cystic from solid masses. However, it is less useful in the evaluation of solid masses. Computed tomography provides excellent anatomical evaluation, can differentiate cystic from solid tumors but has the disadvantage of exposing the child to ionizing radiation and the potential need for sedation. MRI provides better spatial and soft tissue characterization, and without ionizing radiation; nevertheless, it is more costly and takes longer times and is more likely to require sedation [4].

Fine needle aspiration (FNA) can help differentiate inflammatory and neoplastic lesions [5]. In adults, FNA is well established with reported overall accuracy of up to 88% [6]. Nevertheless, its use is controversial in the diagnosis of parotid tumors in children and is thought to have a lower diagnostic yield [7]. It is less tolerable and may require sedation, especially in younger children [5]. Some authors advocated FNA in older children since they are more likely to present with solid and epithelial tumors than younger children who are more likely to present with inflammatory and congenital masses [8]. In our study, FNA had a modest sensitivity of 62.5%, specificity of 50%, and an overall accuracy of 58.4%. Vedrine et al. reported a low FNA accuracy of 33% in children [9]. Low accuracy may be attributed to the difficulty of obtaining FNAs in children and the wide spectrum of possible diagnoses. We advocate FNA in the pediatric population in cases of solid tumors and those that are not clearly congenital or inflammatory on imaging.

In the present study, the most common benign lesion was pleomorphic adenoma (40%), with the rest being variable, including inflammatory and congenital lesions and other benign neoplasms (Table 1). This is consistent with previous similar studies and indicates the more widely variable benign parotid pathologies encountered in the pediatric population when compared to adults [2,3]

**Figure 1:**
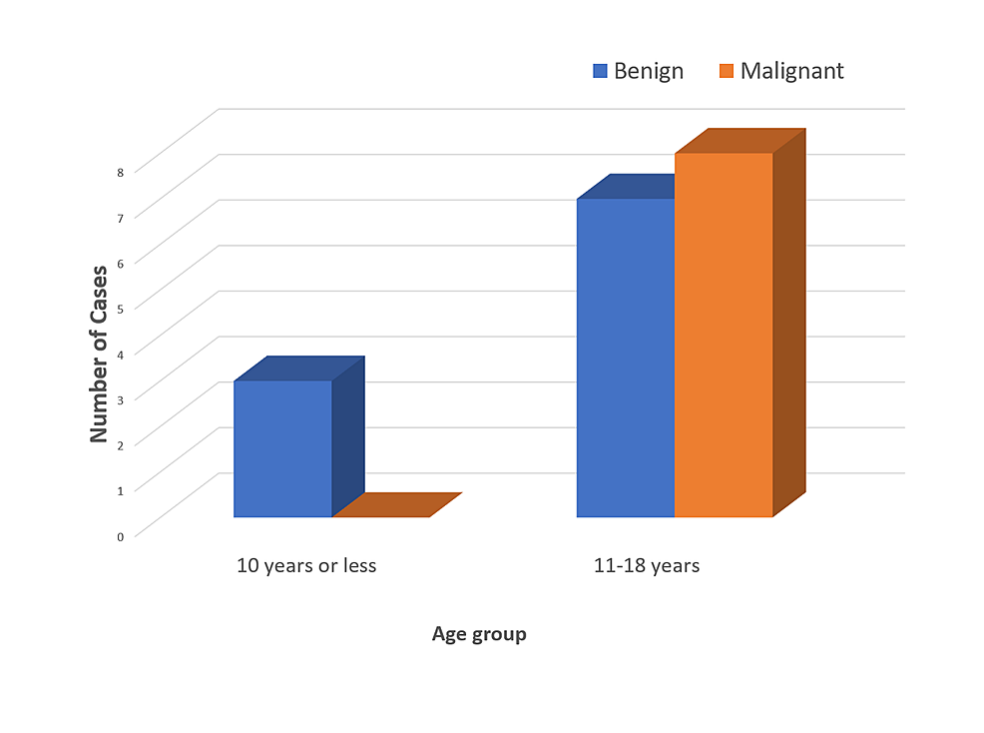
Distribution of pediatric parotid lesions by age and type of pathology

Furthermore, there were eight cases of malignant parotid lesions amounting to 44% of all cases and 53% of those presenting with a solid tumor (Table 1). This is in agreement with previous studies reporting the parotid malignancy rate in the pediatric age group to be more than double that in adults [2,8]. In addition, all of our malignant cases presented in the second decade of life with an age range of 12-17 years. In fact, eight out of 14 cases that presented at this age range were malignant, constituting 57% of all cases presenting in this age range (Figure 1). This is in line with findings from previous studies in other populations reporting that it is unlikely for a parotid tumor presenting at age less than 10 years to be malignant; however, it is more likely to be malignant than benign when presenting in the second decade of life [2,8,10,11].

Mucoepidermoid carcinoma (MEC) was the most common malignancy in our study, which is in line with findings from other studies [10,11]. Nevertheless, five out of six cases of MEC (83%) in our series were high grade (HG MEC). In a recent systemic review of salivary gland malignancies in children by Louredo et al., most parotid MEC were low grade (LG MEC) [11]. Moreover, they reported differences in the epidemiology and pathology of pediatric salivary gland malignancies among different countries [11]. We don’t know if this high rate of HG MEC in our study is by chance or due to bias inherent to tertiary care cases or is reflective of a true higher incidence of HG MEC in our pediatric population. Further larger studies are needed to explore these differences in our population. Moreover, in our series, all epithelial malignancies were MEC. There were no cases of other epithelial malignancies such as adenoid cystic carcinoma, adenocarcinoma, or acinic cell carcinoma, which is reported to be the second most common after MEC [10,11]. We speculate this may be related to our small number of cases. Additionally, the two cases of lymphoma in our study, although not an established indication for parotidectomy, were included because they had undergone formal parotidectomy possibly because of clinical suspicion of an epithelial malignancy.

Adjuvant radiotherapy (RT) in the pediatric age group remains controversial considering their expected long life span and RT-related long-term sequelae such as facial growth retardation, trismus, and radiation-induced malignancies [2,8,10,11]. Some authors recommend postoperative RT for high-grade malignancies, microscopic residuals, aggressive histological features, and lymph node metastasis [2,11]. In their systematic review, Louredo et al. found that adjuvant RT carries a survival benefit in children [11]. To minimize RT-related long-term sequela, conformal radiation therapy techniques, such as intensity-modulated radiotherapy (IMRT), should be used in children whenever possible. IMRT delivers a concentrated radiation dose to the tumor while minimizing the dose to the surrounding healthy tissue [2,5]. No recurrences of MEC were recorded in our patients with a mean follow-up of 58.8 months (Table 2). Furthermore, we had only six cases of MEC, out of which four received adjuvant radiotherapy (RT). Therefore, it is impossible for us to draw conclusions regarding the benefits of RT in limiting recurrences. 

In this study, we had only one inadvertent permanent facial nerve weakness of the marginal branch (5.5%). The rate of temporary facial weakness in our study was 33%. Previous studies on both adult and pediatric parotidectomies reported similar rates ranging from 9.3% to 64.6% [3,12-14]. Some authors suggested a possible higher rate of facial weakness in children [2]. Contrarily, in a previous study by the primary author of the present study, age was not a risk factor for temporary or permanent facial weakness [14]. Similarly, Owusu et al. concluded that age was not predictive of facial weakness in their review of 43 pediatric parotidectomies [13]. Nevertheless, we suggest that parotidectomies in children be performed by surgeons who are familiar with pediatric parotid lesions and anatomy. This is especially important in infants in which facial nerve is very small and attains a more superficial location due to underdeveloped mastoid process [2,13].

Finally, the present study has its limitations. Its retrospective nature makes it limited to available documentation and prone to reporting bias. The patient population was limited to tertiary care centers; thus, selection bias should be considered.

## Conclusions

Parotidectomy was uncommon in the pediatric age group, and its indications were more variable than those in adults. These findings call for a careful preoperative radiological and pathological work-up. We found that pleomorphic adenoma was the most common benign lesion requiring parotidectomy in children, while the most common malignant lesion was mucoepidermoid carcinoma. The rate of high-grade mucoepidermoid carcinoma in our pediatric population was higher than rates reported in previous studies from other populations. However, further studies with larger numbers are needed to confirm this difference. We found that children with solid parotid tumors had a higher risk of parotid malignancy when compared to adults. Furthermore, parotid tumors presenting in the second decade of life should be taken seriously as they were more likely to be malignant than benign.
